# Supplementary material for: Quantitative Proteomics and Differential Protein Abundance Analysis after Depletion of Putative mRNA Receptors in the ER Membrane of Human Cells Identifies Novel Aspects of mRNA Targeting to the ER
Source: Molecules. 2021 Jun 11;26(12):3591. doi: 10.3390/molecules26123591 (PMC8230838; doi:10.3390/molecules26123591)
Supplement: Supplementary file 1 [file molecules-26-03591-s001.zip › Description of Supplementary Tables..pdf]

Description of Supplementary Tables

File Name: Table S1_RRBP1_all.xlsx

Description: Complete list of genes corresponding to proteins quantified in both RRBP1 depletion experiments. Gene names, protein accession numbers (ID), log2 fold changes resulting from siRNA-mediated RRBP1 depletion, and -log10 p values are indicated. Minus sign in front of fold change denotes negatively affected proteins. The number of listed proteins differs from the total number of quantified proteins because some proteins were quantified in less than two of the triplicates. The original Orbitrap data for all quantified proteins are deposited at Proteome Exchange: http://www.proteomexchange.org.

File Name: Table S2_RRBP1_full_lo.xlsx

Description: Proteins that were negatively affected by RRBP1 depletion, i.e. putative RRBP1 clients. Gene names, protein accession numbers, and log2 fold changes resulting from siRNA-mediated RRBP1 depletion are presented together with full protein names and Gene Ontology (GO) annotations for subcellular location(s), as extracted from UniProtKB entries using custom scripts. Proteins are listed according to decreasing negative effects of RRBP1 depletion. In addition, the number of ATTTA motifs in 3′UTRs of the respective mRNAs according to AREsite2 (http://rna.tbi.univie.ac.at/AREsite2/welcome last accessed 02.05.2021) are shown.

File Name: Table S3_RRBP1_full_up.xlsx

Description: Proteins that were positively affected by RRBP1 depletion. Gene names, protein accession numbers, and log2 fold changes resulting from siRNA-mediated RRBP1 depletion are presented together with Gene Ontology (GO) annotations for subcellular location(s), presence of N-terminal signal peptide (SP) or N-terminal transmembrane helix (TMH), total number of transmembrane domains (TMD), number of N-glycosylation sites (Glycosylation sites), amino acid sequences of SP or TMH (in single letter code), TMH position within the total amino acid sequences,

all as extracted from UniProtKB entries using custom scripts. Proteins are listed according to decreasing positive effects of RRBP1 depletion.

File Name: Table S4_KTN1_all.xlsx

Description: Complete list of genes corresponding to proteins quantified in both KTN1 depletion experiments. Gene names, protein accession numbers (ID), log2 fold changes resulting from siRNA-mediated KTN1 depletion, and -log10 p values are indicated. Minus sign in front of fold change denotes negatively affected proteins. The number of listed proteins differs from the total number of quantified proteins because some proteins were quantified in less than two of the triplicates. The original Orbitrap data for all quantified proteins are deposited at Proteome Exchange: http://www.proteomexchange.org.

File Name: Table S5_KTN1_full_lo.xlsx

Description: Proteins that were negatively affected by KTN1 depletion, i.e. putative KTN1 clients. Gene names, protein accession numbers, and log2 fold changes resulting from siRNA-mediated KTN1 depletion are presented together with full protein names and Gene Ontology (GO) annotations for subcellular location(s), as extracted from UniProtKB entries using custom scripts. Proteins are listed according to decreasing negative effects of KTN1 depletion. In addition, the number of ATTTA motifs in 3´UTRs of the respective mRNAs according to AREsite2 (http://rna.tbi.univie.ac.at/AREsite2/welcome last accessed 02.05.2021) are shown.

File Name: Table S6_KTN1_full_up.xlsx

Description: Proteins that were positively affected by KTN1 depletion. Gene names, protein accession numbers, and log2 fold changes resulting from siRNA-mediated KTN1 depletion are presented together with Gene Ontology (GO) annotations for subcellular location(s), presence of N-terminal signal peptide (SP) or N-terminal transmembrane helix (TMH), total number of transmembrane domains (TMD), number of N-glycosylation sites (Glycosylation sites), amino acid sequences of SP or

TMH (in single letter code), TMH position within the total amino acid sequences, all as extracted from UniProtKB entries using custom scripts. Proteins are listed according to decreasing positive effects of KTN1 depletion.

File Name: Table S7_ERj1_all.xlsx

Description: Complete list of genes corresponding to proteins quantified in both ERj1 depletion experiments. Gene names, protein accession numbers (ID), log2 fold changes resulting from siRNA-mediated ERj1 depletion, and -log10 p values are indicated. Minus sign in front of fold change denotes negatively affected proteins. The number of listed proteins differs from the total number of quantified proteins because some proteins were quantified in less than two of the triplicates. The original Orbitrap data for all quantified proteins are deposited at Proteome Exchange: http://www.proteomexchange.org.

File Name: Table S8_ERj1_full_lo.xlsx

Description: Proteins that were negatively affected by ERj1 depletion, i.e. putative ERj1 clients. Gene names, protein accession numbers, and log2 fold changes resulting from siRNA-mediated ERj1 depletion are presented together with full protein names and Gene Ontology (GO) annotations for subcellular location(s), as extracted from UniProtKB entries using custom scripts. Proteins are listed according to decreasing negative effects of ERj1 depletion. In addition, the number of ATTTA motifs in 3´UTRs of the respective mRNAs according to AREsite2 (http://rna.tbi.univie.ac.at/AREsite2/welcome last accessed 02.05.2021) are shown.

File Name: Table S9_ERj1_full_up.xlsx

Description: Proteins that were positively affected by ERj1 depletion. Gene names, protein accession numbers, and log2 fold changes resulting from siRNA-mediated ERj1 depletion are presented together with Gene Ontology (GO) annotations for subcellular location(s), presence of N-terminal signal peptide (SP) or N-terminal transmembrane helix (TMH), total number of transmembrane

domains (TMD), number of N-glycosylation sites (Glycosylation sites), amino acid sequences of SP or TMH (in single letter code), TMH position within the total amino acid sequences, all as extracted from UniProtKB entries using custom scripts. Proteins are listed according to decreasing positive effects of ERj1 depletion.

File Name: Table S10_negatively affected proteins.xlsx

The table summarizes the gene names for proteins, which were negatively affected by the indicated depletions (the putative clients), together with some key features for these proteins, such as presence of SP or TMH and numbers of transmembrane domains (TMD) and N-glycosylation sites. In addition, the concentrations of these proteins in HeLa cells are given. We note that in the case of Sec61 depletion only those proteins are listed, which were also affected in at least one additional depletion. KO refers to CRISPR HEK293 cells, CDG to TRAP deficient fibroblasts from human patients, suffering from congenital disorder of glycosylation.

File Name: Table S11_positively affected proteins.xlsx

The table summarizes putative compensatory mechanisms, i.e. the gene names for proteins, which were positively affected by the indicated depletions and are related to protein biogenesis, together with their functions.

File Name: Table S12_AREs in 3′UTRs of ARE containing genes, coding for negatively affected proteins after depletion of RRBP1, KTN1 and ERj1, respectively.

File Name: Table S13_proteomeXchange_identifiers.docx

The table gives the dataset identifiers for the novel datasets.
