# Supplementary material for: Quantitative Proteomics and Differential Protein Abundance Analysis after Depletion of Putative mRNA Receptors in the ER Membrane of Human Cells Identifies Novel Aspects of mRNA Targeting to the ER
Source: Molecules. 2021 Jun 11;26(12):3591. doi: 10.3390/molecules26123591 (PMC8230838; doi:10.3390/molecules26123591)

Original blots, Figure 5

RM, rough microsomes, were used for comparison
Molecular mass markers are indicated

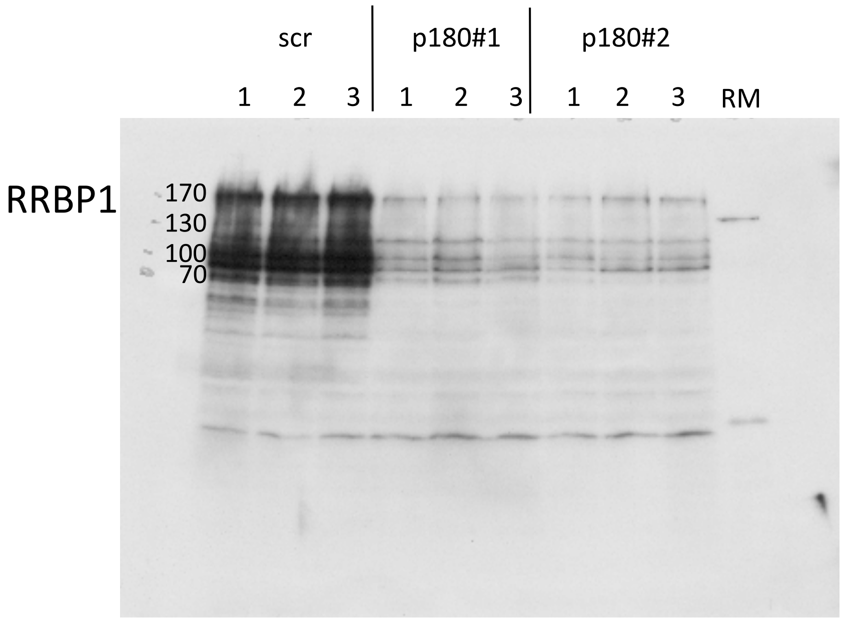

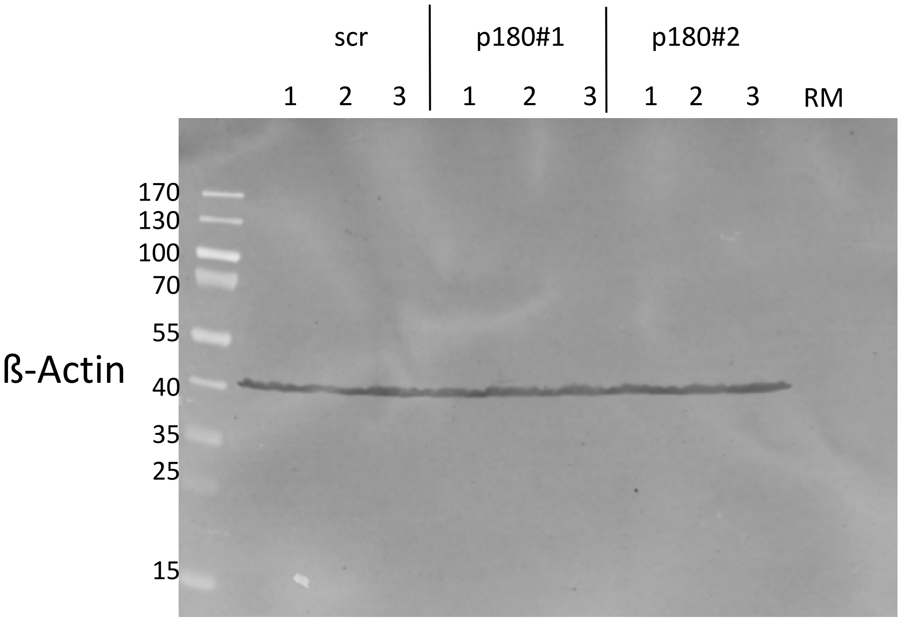

Original blots, Figure 6

RM, rough microsomes, were used for comparison
Molecular mass markers are indicated

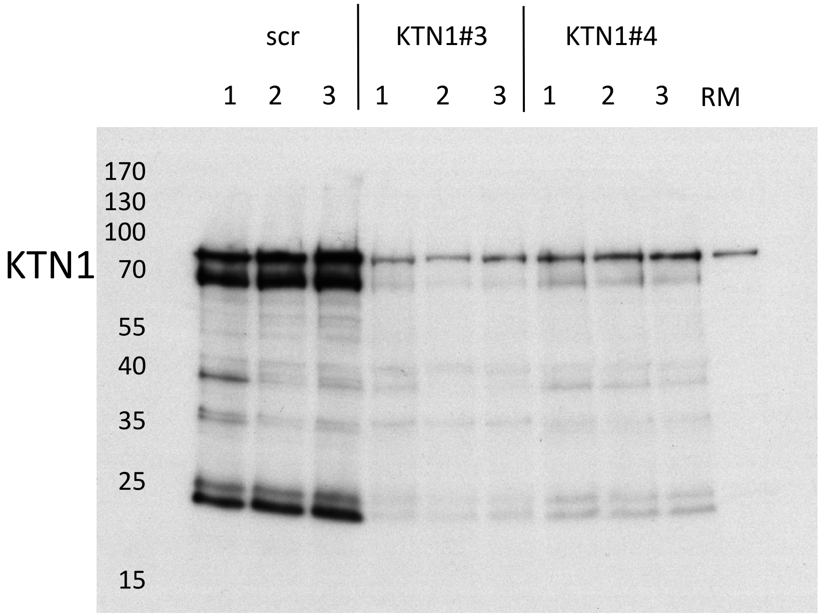

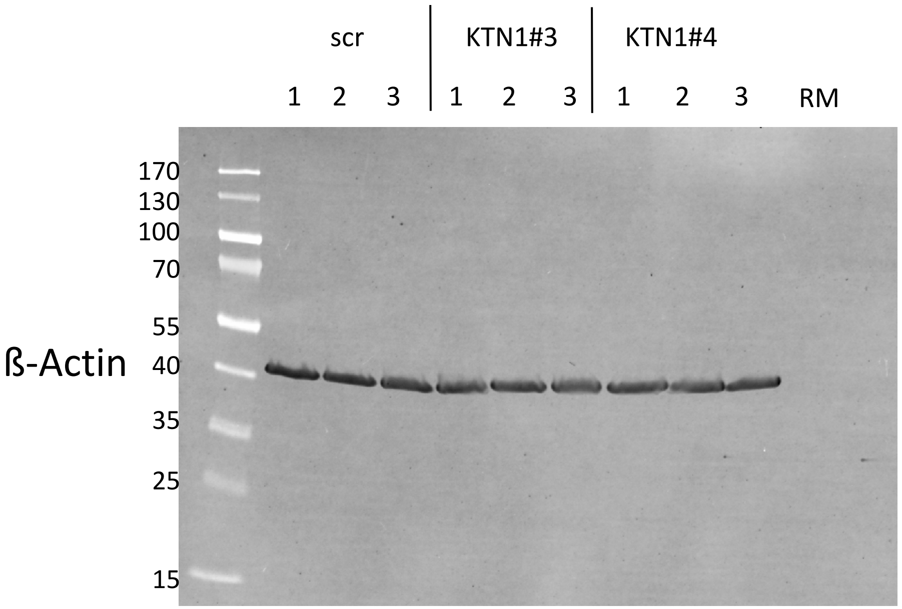

Original blots, Figure 7

RM, rough microsomes, were used for comparison
Molecular mass markers are indicated

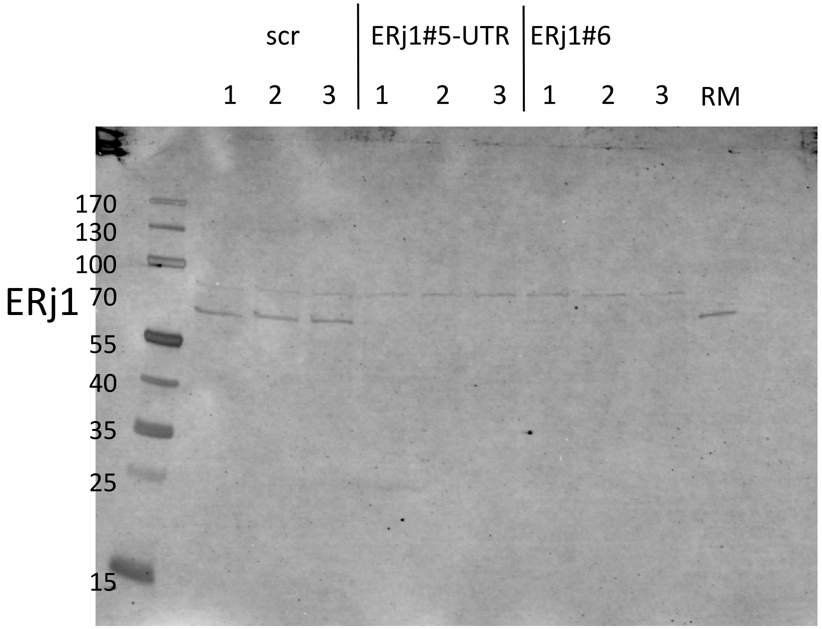

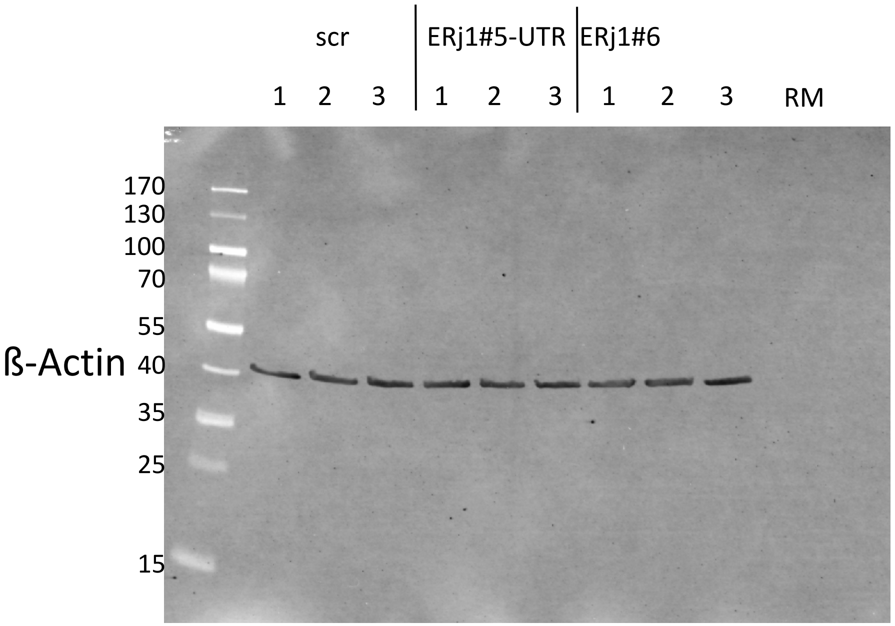

Supplement: Supplementary file 1 [file molecules-26-03591-s001.zip › original blots.pdf]
