# Supplementary material for: Quantitative Proteomics and Differential Protein Abundance Analysis after Depletion of Putative mRNA Receptors in the ER Membrane of Human Cells Identifies Novel Aspects of mRNA Targeting to the ER
Source: Molecules. 2021 Jun 11;26(12):3591. doi: 10.3390/molecules26123591 (PMC8230838; doi:10.3390/molecules26123591)
Supplement: Supplementary file 1 [file molecules-26-03591-s001.zip › Table S13_proteomeXchange_identifiers.pdf]

The mass spectrometry proteomics data (.raw and .txt files) have been deposited to the ProteomeXchange Consortium via the PRIDE partner repository with the dataset identifiers: PXD011989 and PXD011990 (http://www.proteomexchange.org).

Orbi2288	mRNA targeting to the human ER	PXD011989
Sample 1-3	scr control siRNA	
Sample 4-6	P180 siRNA #1	
Sample 7-9	P180 siRNA #2	

Orbi2314	mRNA targeting to the human ER	PXD011990
Sample 1-3	scr control siRNA	
Sample 4-6	KTN1 siRNA #3	
Sample 7-9	KTN1 siRNA #4	

Orbi2314	ERJ1 experiment	PXD011990
Sample 1-3	scr control siRNA	
Sample 10-12	ERJ1 UTR siRNA #4	
Sample 13-15	ERJ1 siRNA #6	
